# Time, Monetary and Other Costs of Participation in Family-Based Child Weight Management Interventions: Qualitative and Systematic Review Evidence

**DOI:** 10.1371/journal.pone.0123782

**Published:** 2015-04-08

**Authors:** Lisa Arai, Monica Panca, Steve Morris, Katherine Curtis-Tyler, Patricia J. Lucas, Helen M. Roberts

**Affiliations:** 1 School of Health and Social Care, Teesside University, Borough Road, Middlesbrough, United Kingdom; 2 Research Department of Primary Care and Population Health, University College London, Rowland Hill Street, London, United Kingdom; 3 Department of Applied Health Research, University College London, 1–19 Torrington Place, London, United Kingdom; 4 School of Health Sciences, City University London, Northampton Square, London, United Kingdom; 5 School for Policy Studies, University of Bristol, Bristol, United Kingdom; 6 University College London Institute of Child Health, London, United Kingdom; Kingston University London, UNITED KINGDOM

## Abstract

**Background:**

Childhood overweight and obesity have health and economic impacts on individuals and the wider society. Families participating in weight management programmes may foresee or experience monetary and other costs which deter them from signing up to or completing programmes. This is recognised in the health economics literature, though within this sparse body of work, costs to families are often narrowly defined and not fully accounted for. A societal perspective incorporating a broader array of costs may provide a more accurate picture. This paper brings together a review of the health economics literature on the costs to families attending child weight management programmes with qualitative data from families participating in a programme to manage child overweight and obesity.

**Methods:**

A search identified economic evaluation studies of lifestyle interventions in childhood obesity. The qualitative work drew on interviews with families who attended a weight management intervention in three UK regions.

**Results:**

We identified four cost-effectiveness analyses that include information on costs to families. These were categorised as direct (e.g. monetary) and indirect (e.g. time) costs. Our analysis of qualitative data demonstrated that, for families who attended the programme, costs were associated both with participation on the scheme and with maintaining a healthy lifestyle afterwards. Respondents reported three kinds of cost: time-related, social/emotional and monetary.

**Conclusion:**

Societal approaches to measuring cost-effectiveness provide a framework for assessing the monetary and non-monetary costs borne by participants attending treatment programmes. From this perspective, all costs should be considered in any analysis of cost-effectiveness. Our data suggest that family costs are important, and may act as a barrier to the uptake, completion and maintenance of behaviours to reduce child obesity. These findings have implications for the development and implementation of child weight initiatives in particular, in relation to reducing inequalities in health.

## Background

### Childhood overweight and obesity

Childhood overweight and obesity carry significant costs to individuals, families and wider society. Obesity in children is a major public health concern, with obese children and adolescents at increased risk of health and social problems in childhood and as adults [1].The global prevalence of obesity is increasing with an estimated 42 million children under five estimated to be overweight or obese in 2010 [2]. The National Child Measurement Programme in England recently found that nearly 19% of children aged 10–11 were obese and a further 14% were overweight. Figures from the Health Survey for England for 2012 show that around 28% of children aged 2 to 15 were overweight or obese [3].

The economic costs of obesity are significant. A systematic review of 32 articles published between 1990 and 2009 estimated that obesity accounted for between 0.7% and 2.8% of an average country’s total healthcare expenditure. Obese individuals were found to have medical costs approximately 30% greater than their normal weight peers [4]. Figures from the USA suggest that childhood obesity imposes a cost to the health system of around US$14 billion per annum [5]. In England, the costs of overweight and obesity to the health system have been calculated to be £4.2 billion per annum. Estimates of the indirect costs (costs to the wider economy, such as loss of productivity) were £15.8 billion [6]. Figures for England suggest that an obese child living in London is likely to cost the health system £31 per year, increasing to £611 per year if their obesity continues into adulthood [7].

### Interventions to address childhood overweight and obesity

There is growing interest in interventions to reduce the prevalence of childhood overweight and obesity and—given demands on health care services—increased interest in the cost-effectiveness of interventions to ascertain whether these interventions represent good value for money.

When assessing the cost-effectiveness of public health interventions, a societal approach to measuring costs [8] may be more appropriate than a narrower public sector or NHS perspective [9]. A societal perspective incorporates the costs and benefits experienced by the individual receiving the treatment into the analysis. These individual level factors are often overlooked within public sector analyses, but can be significant and include aspects of treatment, such as risk preference and patient burden [8]. These can be difficult to measure but their inclusion is likely to strengthen the analysis since, by including costs and benefits experienced by ‘treatment’ recipients, ‘…the harms and benefits associated with undergoing the treatment are included in the results’ ([10], p. 300).

When evaluating the cost and cost-effectiveness of health care programmes to reduce child overweight and obesity, health economics studies tend not to take account of the costs, financial and otherwise, carried by participating families. This may be because they are harder to measure (especially where they are psychological or emotional costs) or because they are considered unimportant if a health service or public sector perspective is taken [8]. Yet such costs can be significant and affect a family’s decision to sign up or, once signed up, to complete a programme. Societal perspectives on the cost-effectiveness of interventions provide a framework for assessing the monetary and non-monetary costs borne by participants attending treatment programmes. From this perspective, *all* costs should be identified and entered into the analysis of cost-effectiveness.

The aim of this paper is to describe different kinds of costs—monetary and otherwise—incurred by families participating in a child weight management programme. In doing so, we contextualise qualitative data drawn from an empirical study [11, 12, 13] with findings from a review of the health economics literature and consider the implications of these for decision making by those developing or implementing child weight management programmes.

### Costs in the health economics literature

An online literature search was performed using PubMed and the NHS Economic Evaluation Database [NHS EED] to identify economic evaluation studies of lifestyle interventions in childhood obesity. To be included in the review studies had to (1) evaluate a family-based lifestyle intervention programme (2) in relation to overweight and/or obese children, and (3) investigate the impact of the programme on costs borne by parents/families. Search terms used in combination with ‘child/childhood obesity’ were “lifestyle intervention”, “parent”, “family”, “cost”, “cost-effectiveness”, “economic evaluation”.

Searches were limited by date from 1990 until April 2014 and to articles available in the English language. Additional studies were identified using reference lists and PubMed related citations.

**Fig 1:**
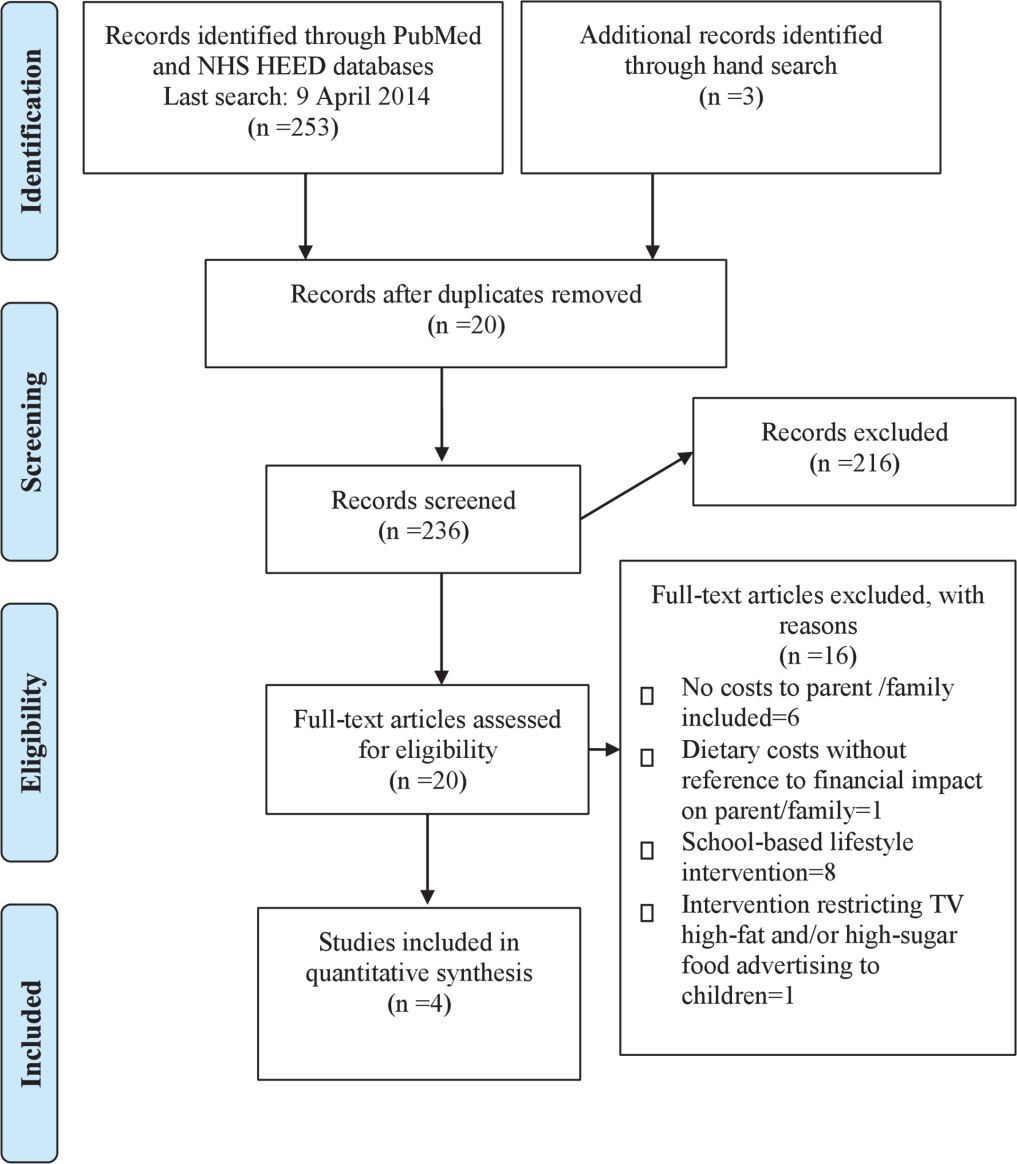
PRISMA flow chart.

The search of PubMed and the NHS EED provided a total of 256 citations (Fig 1). After adjusting for duplicates, 236 remained. Of these, 216 studies were discarded after reviewing abstracts, as they did not meet our inclusion criteria. After the full texts of the remaining 20 were reviewed in detail, eight were excluded because they were school-based programmes, one involved banning television advertisements for energy-dense, nutrient-poor food and beverages during children's peak viewing times, and one reported only dietary costs without reference to financial impact on parent/family. Six lifestyle intervention RCTs were excluded because they focused only on the impact of child obesity on costs incurred by the healthcare systems; costs borne by parents/the family were not included (see S1 Table for a summary of the reasons for exclusion).

We identified four papers reporting findings from three studies. Three fully met our inclusion criteria; the fourth (Banks et al, 2012 [19]), whilst not a family-based intervention itself, was designed to explore cost-barriers to such an intervention, and thus merited inclusion (Table 1.).

**Table 1:** Overview of studies meeting inclusion criteria and authors’ assessment of the impact of the intervention.

Included studies	Interventions	Authors’ assessment of the impact of the intervention
Moodie et al. (2008) [16]	These studies modelled the LEAP (Live, Eat and Play) trial, an intervention targeting behaviour/ lifestyle changes. Participant families in the intervention group received 4 standard GP consultations over 12 weeks; control families received ‘usual care’.	The authors in the Moodie et al. study used modelling techniques to conclude that ‘under current assumptions’ the intervention was cost-effective in terms of disease costs and health benefits, although the uncertainty intervals were wide. The key question was whether the short-term effect (9 months) of the small incremental weight loss remains sustainable over longer period.
Wake et al. (2008)[17]		
	Assumptions were varied from the trial to reflect the real-life situation or implications of repeating the intervention.	The Wake et al. study reported that the intervention resulted in higher costs to families and the health care sector, although, at 15 months, the adjusted BMI or daily physical activity scores did not differ significantly in the intervention group when compared with the control group, but dietary habits had improved.
		The authors conclude that the additional costs to families and the health care sector together with no improvement in outcomes suggest that resources might be better deployed to other uses to create benefit to health and/or wellbeing.
Robertson et al. (2012) [18]	This was a 2 year ‘before-and–after’ evaluation of the 12 week Families for Health intervention, a family based programme for the treatment of childhood obesity. The intervention combined elements from parenting, school-based emotional development and family lifestyle programmes. There was no control group.	The authors conclude that the costs of the programme of £517 per family and the cost per unit change in BMI z-score of £2543 at the 2 year follow-up appeared to be in line with other family-based childhood obesity interventions.
		Improvements and sustainability of BMI z-scores at 2 years lead the authors to conclude that the programme could be a promising childhood intervention.
Banks et al. (2012) [19]	The authors used food diary data provided by families randomized to a clinical trial comparing hospital and primary care childhood obesity clinics. The study explored if healthy eating incurs additional costs when food is purchased in different kinds of shop, and if these costs represent an economic barrier for families with obese children.	The authors conclude that, while for many obese children the costs of eating healthily would not necessarily incur additional costs, a poor diet from a budget supermarket remains the cheapest option. As the poorest families may purchase their food in these outlets, cost may be a barrier.

We provide both a PRISMA checklist (S1 Fig) [14], normally used to demonstrate transparency and completeness of reviews of health care interventions, and the Drummond checklist [15] used for assessing economic evaluations (Table 2.).

**Table 2:** Quality assessment of included studies based on Drummond et al (1997).

Quality assessment		Moodie et al (2008)	Wake et al (2008)	Robertson et al (2012)	Banks et al (2012)
1	Was a well-defined question posed in answerable form?	Yes	Yes	Yes	Yes
2	Was a comprehensive description of the competing alternatives given (i.e. can you tell who did what to whom, where, and how often)?	Yes	Yes	Yes	N/A
3	Was the effectiveness of the programme or services established?	Yes	Yes	Yes	N/A
4	Were all the important and relevant costs and consequences for each alternative identified?	Yes	Yes	Partially	Yes
5	Were costs and consequences measured accurately in appropriate physical units (e.g. hours of nursing time, number of physician visits, lost work-days, gained life years)?	Partially	Partially	Partially	Yes
6	Were the cost and consequences valued credibly?	Yes	Yes	Yes	Yes
7	Were costs and consequences adjusted for differential timing?	Yes	N/A	No	N/A
8	Was an incremental analysis of costs and consequences of alternatives performed?	Yes	No	No	N/A
9	Was allowance made for uncertainty in the estimates of costs and consequences?	Yes	Partially	Partially	N/A
10	Did the presentation and discussion of study results include all issues of concern to users?	Yes	Yes	Yes	Yes

Two Australian papers were identified. The first of these [16] assessed the societal impact of a family GP-based counselling programme for overweight and moderately obese children, modelled on the LEAP (Live, Eat and Play) trial. The study reported that the majority of the costs (78%) were incurred by the health sector and that the costs incurred by children and their families were significant (~21% of total cost of intervention); the family cost components were parents’/children’s time (44.5% of the family cost) and the cost of travelling to and from the programme (55.5% of the family cost). The study did not include family costs incurred in engaging in increased physical activity and costs associated with change in dietary habits.

The second report [17] estimated the resource use of the LEAP programme using an audit of medical records to estimate primary care resource utilisation and questionnaires (at 9 and 15 months) to estimate parent-reported family resource use. The results showed that the cost per intervention family was AU $4094 greater than per control family because of resources devoted to child physical activity. Although there were differences between groups when averages of different type of costs were reported (e.g., parents spent ~33% more for sport/physical activity purchases over 15 months in intervention group), these differences were not statistically significant. The authors reported that there were statistically significant differences in adult time helping children to be physically active (7.8 vs. 4.9 hours/week for intervention families, representing ~37% more adult time cost per week for the first 9 months; this additional time spent was not sustained, and became similar to the control families over the following 6 month period, 4.8 hours vs. 5.3 hours/week). Differences between groups in terms of time and monetary values invested by families in changing dietary habits were also reported as not statistically significant. Weekly expenses and time costs over 15 months were ~7% (p = 0.03) greater for intervention families.

The other two papers reported findings from two UK-based studies. One [18] evaluated the two-year follow-up of the ‘Families for Health’ programme for the treatment of childhood obesity. The study recorded the costs to deliver the intervention and family-reported resource use (in terms of time and monetary values to attend the programme, change in dietary habits, investments in new clothes and child care) based on questionnaires. The direct cost of the programme was £517 per family, including both the health care and the family sector. Direct costs for families represented ~11% of the total cost of the programme and were attributable to travel cost (59% of the family cost) and food/clothes expenses (41% of the family cost). Indirect costs were not expressed in monetary terms only as amount of time parents needed to attend the programme (on average 33h per family). It was reported that one mother gave up paid employment and that four other families needed to provide alternative care (unpaid carers) for their younger children while attending the programme.

The second UK study [19] assessed the costs of dietary change and the impact on food shopping patterns for families with obese children. The study theoretically adjusted a 3-day dietary diary completed by parents of obese children to meet the estimated average requirements for each participant and currently healthy eating guidelines promoted by “Eatwell plate”. The researchers concluded that switching to healthier alternative menus could increase the cost by 33 pence/day if purchased at the budget supermarket, four pence/day if purchased at a mid-range supermarket and lower the cost by 17 pence/day if purchased on the high street. Switching from an unhealthy menu purchased in a mid-range supermarket to a healthier alternative purchased in a budget supermarket could save 59 pence/day. Budget-outlet shops are also a cheaper alternative to mid-range supermarkets (59 pence/day saved for the same healthier menu purchased). The authors concluded that the cost of healthier alternative could be a barrier in change of dietary habits for poor families.

The heterogeneity of cost components, type of lifestyle interventions, age of the children participating and methodologies used to obtain final results did not allow an overall synthesis of the data reported in these studies. However, across the four studies, costs to families could be either direct (e.g. monetary) or indirect (e.g. time) and could be associated with either taking the child to the programme and/or helping the child attain and maintain a healthy weight.

Below, we explore the scope and type of costs incurred by families drawing on qualitative data from a mixed methods evaluation of a family-based intervention to address overweight and obesity in children initially shown to be useful in a trial [11, 12, 13], and then rolled out at scale.

## Materials and Methods

Our sampling strategy for the qualitative data aimed to maximise variation across multiple deprivation at the small area level, family structure, parent-reported participant ethnicity, housing tenure and completion status of a weight management programme. In addition, we purposively selected two rural families. Full methodological details are available elsewhere [11, 12, 13].

The data were collected in three locations in England (North East, South West and London). A sample of families who had participated in a weight management programme were interviewed and asked about their experience of attending the scheme (N = 23). The family groups varied in size and composition, but every group contained at least one adult and one child. Some groups were multi-generational, with children, parents and grandparents present. Respondents were asked about referral to the intervention, their experiences on the programme, weight loss during and after participation and any costs they had incurred. ‘Costs’ were not defined but the researchers indicated that these could be monetary or non-monetary. The qualitative dataset was analysed using Framework analytic techniques [20].

### Ethics Statement

UCL Ethics Committee granted approval for this study in February 2011 (REF: 2842/001). We conducted the project with reference to the framework for research ethics produced by the Economic and Social Research Council. Written informed consent was obtained from all participants. The interview material reported here is identified only by a family (F) number, and whether a child, mother or father is speaking.

## Results

For the families who attended the programme, costs were associated with participation and maintaining a healthy lifestyle post-intervention. Respondents reported three types of cost which were frequently inter-related: time, social/emotional and monetary. Table 3 illustrates these.

**Table 3:** Costs described by families.

Costs described by families	
Time-related costs	‘Time probably more so than the actual cost… although the cost of it, everything does go up.’ Mother F26 (but see also F26 monetary costs below).
	‘I finish work at 5. . .So I would go and pick them up with sandwiches to eat on the way…By the time you get back, it’s 9 o’clock. It’s way after their bedtime.’ Mother F13
	‘We can’t carry on going. . . .because we’re really busy now, and we go to an Arabic class in the…Mosque, and it’s from five o’clock till seven o’clock.’ Boy, F49
Social/emotional costs	‘…he [father] tended to have a whinge a little bit [about taking his daughter] because he was a contractor and if he takes times off work he doesn’t get paid. So more often than not it was me.’ Mother F21‘Most of my friends they have the unhealthy food in their house and they’re allowed to eat it.’ Boy, F26
	‘It breaks my heart sometimes when I say no to them.’ Mother, F53
	‘The [programme] top, I didn’t like the top…I got out of the car, launched it on, and then launched a coat over it. It was just really embarrassing…’ Boy, F18
	‘I’ll explain to my mum when she uses so much oil I say, ‘Mum, don’t use so much oil because you could use half of that quantity of oil and still make it,’ and she’ll say, ‘Well I’ve had it all my life darling, nothing’s happened to me.’ Mother (F53)
Monetary costs	‘. . .they say buy the lean mince, well, how much is lean mince in [supermarket name] that costs a fortune. Because you can buy their own make but most of it is fat, you put it in a colander, it’s horrible so, what do you do?’ Mother F61
	‘. . .you do have to change your shopping habits. A lot of the time if you are on—this is going to sound awful—on the basic ranges, so things like the basic meats and the value ranges, they tend to be quite high in fats because they are cheaper to produce, so you are then looking at actually going up to the more superior quality to get the low fats and the lower carbohydrates and things like that.’ Father, F4
	‘…they need a swimming costume … then they’ve got to swim. We’ve got to get there, pay the parking, and then the kids will want something to eat when they get out as well.’ Auntie, F57

### Time-related costs

Time-related costs of attendance were primarily about fitting a programme with twice weekly attendance into busy working and family lives and school. Families changed working patterns and shifts in order to attend and sometimes travelled long distances. For one North East England-based family, for example, the nearest programme would have been hard to get to in rush hour so they went to one 16 miles away which meant that the mother had to take time off work (F21). Undertaking an extra activity could be difficult for children with other after-school commitments including religious observance and sports activities. For parents, understanding employers or flexible working hours could help in taking children to the programme, but not all were able to do this.

### Social/emotional costs

The emotional and social costs of participating were rarely identified as such by families. However, it was evident that families had had to make changes during and after the intervention, ones which were related to lack of time and exerted a social or emotional toll. Parents, usually mothers, spoke of the strain on their children. For some children, choosing the healthy option meant being ‘different’ or missing out on other opportunities. The changes needed for success on the programme could create friction between parents and children, exposing fragile relationships and threatening family dynamics. One mother spoke about the damage she felt had been done to her relationship with her daughter by spending her teenage years fighting about food. Her daughter acknowledged that her diet was her choice and responsibility while simultaneously blaming her mother for giving her bad habits and allowing her to make unhealthy choices. Both mother and daughter felt they were to blame. Tensions and contradictions were evident in some family discussions with participants wanting to ‘do the right thing’ in relation to weight management. Aside from differences between respondents in recollection of programme logistics, such as timing and frequency, most differences arose from assertions or defences of moral character, or challenges to someone else’s, in relation to behaviour or weight. For example, one child contested his mother’s claim that they visited the sports centre to check for activities at the beginning of last month, saying that it was more than six months ago (Boy, F14). Several children disagreed with their parents that they had regained weight lost on the programme. More than one Bangladeshi or Pakistani family described moving to what they described as ‘non-Asian’ foods. A perception of ‘Asian’ food as unhealthy implies a change that—in part—rejects cultural norms and identity. This conflict between healthy eating and traditional practices has been noted by others [21] and evoked in observations by both children and mothers talking about the role of grandmothers in supporting or undermining weight management efforts.

### Monetary costs

Respondents were asked if they had incurred monetary costs while participating on the programme or after it had finished. Few explicitly did so. While many of the barriers to attending the programme described by parents would have resulted in costs, they were not usually identified as such by families. Time off work, childcare and, in particular, driving to the venue carried costs but were often described as ‘normal’ costs. Additional costs included trips to the cinema if targets were met, or joining children up to exercise classes. Those who did identify costs usually related them to switching to ‘healthier’ foods, with respondents describing the more expensive, ‘healthier’ food as beyond the reach of families on a low income. Some discussed the fact that low cost ranges and low cost supermarkets do not include the healthier alternatives they had been encouraged to buy while on the programme.

## Discussion

Most analyses of the cost-effectiveness of childhood obesity interventions do not include the costs borne by families during or after participation in the programme. We identified four studies from the health economics literature that did include this detail, and analysis of these showed that, when costs borne by families have been included, these tend to be significant and are largely about time and money.

These findings are supported by qualitative data collected during an evaluation of a family-based child weight management programme. While parents and carers, as well as children often reported enjoying the programme, families struggled to fit it into work and school schedules, or combine it with post-school activities. Respondents also reported emotional and social costs as attempts to maintain new diet or exercise behaviours impacted on family dynamics and relationships, or exposed tensions between family members. These costs could also be incurred or experienced beyond the family; some children and parents reported social embarrassment about attending the scheme, and some parents initially viewed walking through the door as an implicit admission of parental culpability. Whilst families rarely referred to direct monetary costs as a result of being on the scheme, respondents did speak of the higher costs of ‘healthier’ foods and, in some cases, this had deterred them from purchasing these foodstuffs.

### Strengths and limitations

To enhance generalizability and quality, qualitative data were collected in several English locations by experienced qualitative researchers. We recruited a (demographically) broad array of respondents, and attempted to include those who had declined the intervention or had minimal participation. However, the latter proved difficult to achieve and our respondents were largely programme participants who had stayed the course and may, therefore, have incurred fewer costs and experienced more benefits than those who had dropped out or failed to attend even one session. There was, in some cases, a significant time lag between attendance on the programme and the collection of evaluation data, so that some respondents could not accurately recall the scope or type of costs incurred during or after the programme.

In summary, our data suggests that family costs are an important cost component and may act as a barrier to the successful uptake and completion of childhood lifestyle interventions to reduce obesity. We make four observations here aimed at those commissioning and designing child weight management programmes.

First, a societal perspective that utilises direct and measurable, as well as indirect time, social and emotional, costs to families should be the default analytic approach in the assessment of the cost-effectiveness of child weight management programmes. NICE recently produced a costing template to estimate the financial impact to the NHS of implementing guidance on tackling childhood overweight and obesity in England [22] designed to be used to assess the local impact of implementing the recommendations. It (reasonably) focuses on the factors that require the most resources to implement or will generate the most savings. What costing tools of this kind cannot (and are not designed to) do is assess the costs to individuals and families, or the wider community. Assessments of this kind provide an indication of the likely impact and are not absolute figures, but without a better understanding of the hidden costs to families, costings which relate only to service costs may be of limited value. Such analyses are likely to require input from both health economists and qualitative researchers working together.

Second, as with all NICE costings approaches, the national assumptions used in the template described above can be altered to reflect local circumstances. Those working at the service frontline are well-placed to consider this. While it is unlikely that a metric could be produced, this is an area where local ‘know-how’ as a form of knowledge is crucial to help mitigate the type of time, emotional and monetary costs identified in our analysis. The Localism Bill [23] has granted greater powers to local authorities over the commercial provision of food in their areas. Healthier school meals, or a reduction in fast food outlets, may help in the creation of a healthier local food environment—one which has the potential to ameliorate the adverse health consequences of obesogenic urban high streets [24]. A local, healthy food culture may also influence family perceptions of healthy eating, force re-consideration of entrenched dietary habits and reduce the social stigma of attending weight management interventions.

Third, although we refer throughout to families, the time, practical, emotional and culinary work was highly gendered, falling disproportionately to mothers. This is likely to be the case with a whole range of parenting and family-based interventions. Creative ways of mitigating these costs need to be considered, in particular in relation to reducing inequalities in child health.

Finally, there is a socio-economic gap in childhood overweight and obesity. Trial and cost data with relatively short term follow-up may not capture the information needed to successfully implement programmes on a large scale and maintain gains. Data such as those in this paper can provide practical pointers to the kinds of implementation of programme design and delivery that may be more feasible for the most disadvantaged families.

## Supporting Information

S1 Fig PRISMA checklist. (ZIP)

S1 Table Excluded studies. (DOCX)
