# Supplementary material for: Time, Monetary and Other Costs of Participation in Family-Based Child Weight Management Interventions: Qualitative and Systematic Review Evidence
Source: PLoS One. 2015 Apr 8;10(4):e0123782. doi: 10.1371/journal.pone.0123782 (PMC4390145; doi:10.1371/journal.pone.0123782)
Supplement: S1 Table — (DOCX) [file pone.0123782.s002.docx]

**S1 Table: Excluded studies**

| **Reference** | **Type of study** | **Reason for exclusion** |
| --- | --- | --- |
| Kalavainen M, Karjalainen S, Martikainen J, Korppi M, Linnosmaa I, Nuutinen O. Cost-effectiveness of routine and groupprograms for treatment of obesechildren. Pediatr Int. 2009; 51(5):606-11. | Cost-effectiveness analysis | No costs to parent/family included |
| [Janicke DM](http://www.ncbi.nlm.nih.gov/pubmed?term=Janicke%20DM%5BAuthor%5D&cauthor=true&cauthor_uid=19566621), [Sallinen BJ](http://www.ncbi.nlm.nih.gov/pubmed?term=Sallinen%20BJ%5BAuthor%5D&cauthor=true&cauthor_uid=19566621), [Perri MG](http://www.ncbi.nlm.nih.gov/pubmed?term=Perri%20MG%5BAuthor%5D&cauthor=true&cauthor_uid=19566621), [Lutes LD](http://www.ncbi.nlm.nih.gov/pubmed?term=Lutes%20LD%5BAuthor%5D&cauthor=true&cauthor_uid=19566621), [Silverstein JH](http://www.ncbi.nlm.nih.gov/pubmed?term=Silverstein%20JH%5BAuthor%5D&cauthor=true&cauthor_uid=19566621), [Brumback B](http://www.ncbi.nlm.nih.gov/pubmed?term=Brumback%20B%5BAuthor%5D&cauthor=true&cauthor_uid=19566621). Comparison of program costs for parent-only and family-based interventions for pediatric obesity in medically underserved rural settings. [J Rural Health.](http://www.ncbi.nlm.nih.gov/pubmed/?term=Comparison+of+Program+Costs+for+Parent-Only+and+Family-Based+Interventions+for+Pediatric+Obesity+in+Medically+Underserved+Rural+Settings) 2009; 25(3):326-30. | Cost analysis | No costs to parent/family included |
| [Goldfield GS](http://www.ncbi.nlm.nih.gov/pubmed?term=Goldfield%20GS%5BAuthor%5D&cauthor=true&cauthor_uid=11781766), [Epstein LH](http://www.ncbi.nlm.nih.gov/pubmed?term=Epstein%20LH%5BAuthor%5D&cauthor=true&cauthor_uid=11781766), [Kilanowski CK](http://www.ncbi.nlm.nih.gov/pubmed?term=Kilanowski%20CK%5BAuthor%5D&cauthor=true&cauthor_uid=11781766), [Paluch RA](http://www.ncbi.nlm.nih.gov/pubmed?term=Paluch%20RA%5BAuthor%5D&cauthor=true&cauthor_uid=11781766), [Kogut-Bossler B](http://www.ncbi.nlm.nih.gov/pubmed?term=Kogut-Bossler%20B%5BAuthor%5D&cauthor=true&cauthor_uid=11781766). Cost-effectiveness of group and mixed family-based treatment for childhood obesity. [Int J Obes Relat Metab Disord.](http://www.ncbi.nlm.nih.gov/pubmed/?term=Cost-effectiveness+of+group+and+mixed+family-based+treatment+for+childhood+obesity) 2001; 25(12):1843-9. | Cost-effectiveness analysis | No costs to parent/family included |
| [Raynor HA](http://www.ncbi.nlm.nih.gov/pubmed?term=Raynor%20HA%5BAuthor%5D&cauthor=true&cauthor_uid=12008989), [Kilanowski CK](http://www.ncbi.nlm.nih.gov/pubmed?term=Kilanowski%20CK%5BAuthor%5D&cauthor=true&cauthor_uid=12008989), [Esterlis I](http://www.ncbi.nlm.nih.gov/pubmed?term=Esterlis%20I%5BAuthor%5D&cauthor=true&cauthor_uid=12008989), [Epstein LH](http://www.ncbi.nlm.nih.gov/pubmed?term=Epstein%20LH%5BAuthor%5D&cauthor=true&cauthor_uid=12008989). A cost-analysis of adopting a healthful diet in a family-based obesity treatment program. [J Am Diet Assoc.](http://www.ncbi.nlm.nih.gov/pubmed/?term=A+cost-analysis+of+adopting+a+healthful+diet+in+a+family-based+obesity+treatment+program) 2002; 102(5):645-56. | Cost analysis | Dietary costs without reference to financial impact on parent/family: e.g. price variability/access to food shops |
| Wake M, Baur LA, Gerner B, Gibbons K, Gold L, Gunn J, Levickis P, McCallum Z, Naughton G, Sanci L, Ukoumunne OC. [Outcomes and costs of primary care surveillance and intervention for overweight or obese children: the LEAP 2 randomised controlled trial.](http://www.ncbi.nlm.nih.gov/pubmed/19729418) BMJ. 2009 Sep 3; 339:b3308. | Cost-effectiveness analysis | No costs to parent/family included |
| [McAuley KA](http://www.ncbi.nlm.nih.gov/pubmed?term=McAuley%20KA%5BAuthor%5D&cauthor=true&cauthor_uid=19444231), [Taylor RW](http://www.ncbi.nlm.nih.gov/pubmed?term=Taylor%20RW%5BAuthor%5D&cauthor=true&cauthor_uid=19444231), [Farmer VL](http://www.ncbi.nlm.nih.gov/pubmed?term=Farmer%20VL%5BAuthor%5D&cauthor=true&cauthor_uid=19444231), [Hansen P](http://www.ncbi.nlm.nih.gov/pubmed?term=Hansen%20P%5BAuthor%5D&cauthor=true&cauthor_uid=19444231), [Williams SM](http://www.ncbi.nlm.nih.gov/pubmed?term=Williams%20SM%5BAuthor%5D&cauthor=true&cauthor_uid=19444231), [Booker CS](http://www.ncbi.nlm.nih.gov/pubmed?term=Booker%20CS%5BAuthor%5D&cauthor=true&cauthor_uid=19444231), [Mann JI](http://www.ncbi.nlm.nih.gov/pubmed?term=Mann%20JI%5BAuthor%5D&cauthor=true&cauthor_uid=19444231). Economic evaluation of a community-based obesity prevention program in children: the APPLE project. [Obesity (Silver Spring).](http://www.ncbi.nlm.nih.gov/pubmed/?term=Economic+Evaluation+of+a+Community-based+Obesity+Prevention+Program+in+Children%3A+The+APPLE+Project) 2010; 18(1):131-6. | Cost-effectiveness analysis | No costs to parent/family included |
| [Coppins DF](http://www.ncbi.nlm.nih.gov/pubmed?term=Coppins%20DF%5BAuthor%5D&cauthor=true&cauthor_uid=21487425), [Margetts BM](http://www.ncbi.nlm.nih.gov/pubmed?term=Margetts%20BM%5BAuthor%5D&cauthor=true&cauthor_uid=21487425), [Fa JL](http://www.ncbi.nlm.nih.gov/pubmed?term=Fa%20JL%5BAuthor%5D&cauthor=true&cauthor_uid=21487425), [Brown M](http://www.ncbi.nlm.nih.gov/pubmed?term=Brown%20M%5BAuthor%5D&cauthor=true&cauthor_uid=21487425), [Garrett F](http://www.ncbi.nlm.nih.gov/pubmed?term=Garrett%20F%5BAuthor%5D&cauthor=true&cauthor_uid=21487425), [Huelin S](http://www.ncbi.nlm.nih.gov/pubmed?term=Huelin%20S%5BAuthor%5D&cauthor=true&cauthor_uid=21487425). Effectiveness of a multi-disciplinary family-based programme for treating childhood obesity (the Family Project). [Eur J Clin Nutr.](http://www.ncbi.nlm.nih.gov/pubmed/?term=Effectiveness+of+a+multi-disciplinary+family-based+programme+for+treating+childhood+obesity+%28The+Family+Project%29) 2011; 65(8):903-9. | Cost analysis | No costs to parent/family included |
| Wang LY, Yang Q, Lowry R, Wechsler H. [Economic analysis of a school-based obesity prevention program.](http://www.ncbi.nlm.nih.gov/pubmed/14627751) Obes Res. 2003; 11(11):1313-24. | Cost-utility analysis | School-based lifestyle intervention; no costs to parent/family included |
| Wang LY, Gutin B, Barbeau P, Moore JB, Hanes J Jr, Johnson MH, Cavnar M, Thornburg J, Yin Z. [Cost-effectiveness of a school-based obesity prevention program.](http://www.ncbi.nlm.nih.gov/pubmed/19000237) J Sch Health. 2008; 78(12):619-24. | Cost-effectiveness analysis | School-based lifestyle intervention; no costs to parent/family included |
| Brown HS 3rd, Pérez A, Li YP, Hoelscher DM, Kelder SH, Rivera R. [The cost-effectiveness of a school-based overweight program.](http://www.ncbi.nlm.nih.gov/pubmed/17908315) Int J BehavNutr Phys Act. 2007; 4:47. | Cost-effectiveness analysis | School-based lifestyle intervention; no costs to parent/family included |
| [Magnus A](http://www.ncbi.nlm.nih.gov/pubmed?term=Magnus%20A%5BAuthor%5D&cauthor=true&cauthor_uid=19652656), [Haby MM](http://www.ncbi.nlm.nih.gov/pubmed?term=Haby%20MM%5BAuthor%5D&cauthor=true&cauthor_uid=19652656), [Carter R](http://www.ncbi.nlm.nih.gov/pubmed?term=Carter%20R%5BAuthor%5D&cauthor=true&cauthor_uid=19652656), [Swinburn B](http://www.ncbi.nlm.nih.gov/pubmed?term=Swinburn%20B%5BAuthor%5D&cauthor=true&cauthor_uid=19652656). The cost-effectiveness of removing television advertising of high-fat and/or high-sugar food and beverages to Australian children. [Int J Obes (Lond).](http://www.ncbi.nlm.nih.gov/pubmed/19652656) 2009; 33(10):1094-102. | Cost-effectiveness analysis | Intervention restricting TV high-fat and/or high-sugar food advertising to children |
| [Moodie ML](http://www.ncbi.nlm.nih.gov/pubmed?term=Moodie%20ML%5BAuthor%5D&cauthor=true&cauthor_uid=19893504), [Carter RC](http://www.ncbi.nlm.nih.gov/pubmed?term=Carter%20RC%5BAuthor%5D&cauthor=true&cauthor_uid=19893504), [Swinburn BA](http://www.ncbi.nlm.nih.gov/pubmed?term=Swinburn%20BA%5BAuthor%5D&cauthor=true&cauthor_uid=19893504), [Haby MM](http://www.ncbi.nlm.nih.gov/pubmed?term=Haby%20MM%5BAuthor%5D&cauthor=true&cauthor_uid=19893504). The cost-effectiveness of Australia's Active After-School Communities program. [Obesity (Silver Spring).](http://www.ncbi.nlm.nih.gov/pubmed/?term=The+Cost-effectiveness+of+Australia%E2%80%99s+Active+After-school+Communities+Program) 2010; 18(8):1585-92. | Cost-effectiveness analysis | School-based lifestyle intervention; no costs to parent/family included |
| [Moodie M](http://www.ncbi.nlm.nih.gov/pubmed?term=Moodie%20M%5BAuthor%5D&cauthor=true&cauthor_uid=19747402), [Haby M](http://www.ncbi.nlm.nih.gov/pubmed?term=Haby%20M%5BAuthor%5D&cauthor=true&cauthor_uid=19747402), [Galvin L](http://www.ncbi.nlm.nih.gov/pubmed?term=Galvin%20L%5BAuthor%5D&cauthor=true&cauthor_uid=19747402), [Swinburn B](http://www.ncbi.nlm.nih.gov/pubmed?term=Swinburn%20B%5BAuthor%5D&cauthor=true&cauthor_uid=19747402), [Carter R](http://www.ncbi.nlm.nih.gov/pubmed?term=Carter%20R%5BAuthor%5D&cauthor=true&cauthor_uid=19747402). Cost-effectiveness of active transport for primary school children - Walking School Bus program. [Int J BehavNutr Phys Act.](http://www.ncbi.nlm.nih.gov/pubmed/?term=Cost-effectiveness+of+active+transport+for+primary+school+children+-+Walking+School+Bus+program) 2009; 6:63. | Cost-effectiveness analysis | School-based lifestyle intervention; no costs to parent/family included |
| [Moodie M](http://www.ncbi.nlm.nih.gov/pubmed?term=Moodie%20M%5BAuthor%5D&cauthor=true&cauthor_uid=21597123), [Haby MM](http://www.ncbi.nlm.nih.gov/pubmed?term=Haby%20MM%5BAuthor%5D&cauthor=true&cauthor_uid=21597123), [Swinburn B](http://www.ncbi.nlm.nih.gov/pubmed?term=Swinburn%20B%5BAuthor%5D&cauthor=true&cauthor_uid=21597123), [Carter R](http://www.ncbi.nlm.nih.gov/pubmed?term=Carter%20R%5BAuthor%5D&cauthor=true&cauthor_uid=21597123). Assessing cost-effectiveness in obesity: active transport program for primary school children--TravelSMART Schools Curriculum program. [J Phys Act Health.](http://www.ncbi.nlm.nih.gov/pubmed/?term=Assessing+Cost-Effectiveness+in+Obesity%3A+Active+Transport+Program+for+Primary+School+Children%E2%80%94+TravelSMART+Schools+Curriculum+Program) 2011; 8(4):503-15. | Cost-effectiveness analysis | School-based lifestyle intervention; no costs to parent/family included |
| Meng L, Xu H, Liu A, van Raaij J, Bemelmans W, Hu X, Zhang Q, Du S, Fang H, Ma J, Xu G, Li Y, Guo H, Du L, Ma G. [The costs and cost-effectiveness of a school-based comprehensive intervention study on childhood obesity in China.](http://www.ncbi.nlm.nih.gov/pubmed/24205050)PLoS One. 2013; 8(10):e77971. | Cost-effectiveness analysis | School-based lifestyle intervention; no costs to parent/family included |
| [Kesztyüs D](http://www.ncbi.nlm.nih.gov/pubmed?term=Keszty%C3%BCs%20D%5BAuthor%5D&cauthor=true&cauthor_uid=21986721), [Schreiber A](http://www.ncbi.nlm.nih.gov/pubmed?term=Schreiber%20A%5BAuthor%5D&cauthor=true&cauthor_uid=21986721), [Wirt T](http://www.ncbi.nlm.nih.gov/pubmed?term=Wirt%20T%5BAuthor%5D&cauthor=true&cauthor_uid=21986721), [Wiedom M](http://www.ncbi.nlm.nih.gov/pubmed?term=Wiedom%20M%5BAuthor%5D&cauthor=true&cauthor_uid=21986721), [Dreyhaupt J](http://www.ncbi.nlm.nih.gov/pubmed?term=Dreyhaupt%20J%5BAuthor%5D&cauthor=true&cauthor_uid=21986721), [Brandstetter S](http://www.ncbi.nlm.nih.gov/pubmed?term=Brandstetter%20S%5BAuthor%5D&cauthor=true&cauthor_uid=21986721), [Koch B](http://www.ncbi.nlm.nih.gov/pubmed?term=Koch%20B%5BAuthor%5D&cauthor=true&cauthor_uid=21986721), [Wartha O](http://www.ncbi.nlm.nih.gov/pubmed?term=Wartha%20O%5BAuthor%5D&cauthor=true&cauthor_uid=21986721), [Muche R](http://www.ncbi.nlm.nih.gov/pubmed?term=Muche%20R%5BAuthor%5D&cauthor=true&cauthor_uid=21986721), [Wabitsch M](http://www.ncbi.nlm.nih.gov/pubmed?term=Wabitsch%20M%5BAuthor%5D&cauthor=true&cauthor_uid=21986721), [Kilian R](http://www.ncbi.nlm.nih.gov/pubmed?term=Kilian%20R%5BAuthor%5D&cauthor=true&cauthor_uid=21986721), [Steinacker JM](http://www.ncbi.nlm.nih.gov/pubmed?term=Steinacker%20JM%5BAuthor%5D&cauthor=true&cauthor_uid=21986721). Economic evaluation of URMEL-ICE, a school-based overweight prevention programme comprising metabolism, exercise and lifestyle intervention in children. [Eur J Health Econ.](http://www.ncbi.nlm.nih.gov/pubmed/?term=Economic+evaluation+of+URMEL-ICE%2C+a+school-based+overweight+prevention+programme+comprising+metabolism%2C+exercise+and+lifestyle+intervention+in+children) 2013 Apr; 14(2):185-95. | Cost-effectiveness analysis | School-based lifestyle intervention; no costs to parent/family included |
